# Supplementary figures and images for: Mycobacterium tuberculosis-specific CD4+ and CD8+ T cells differ in their capacity to recognize infected macrophages
Source: PLoS Pathog. 2018 May 21;14(5):e1007060. doi: 10.1371/journal.ppat.1007060 (PMC6013218; doi:10.1371/journal.ppat.1007060)

S1 Fig

A.

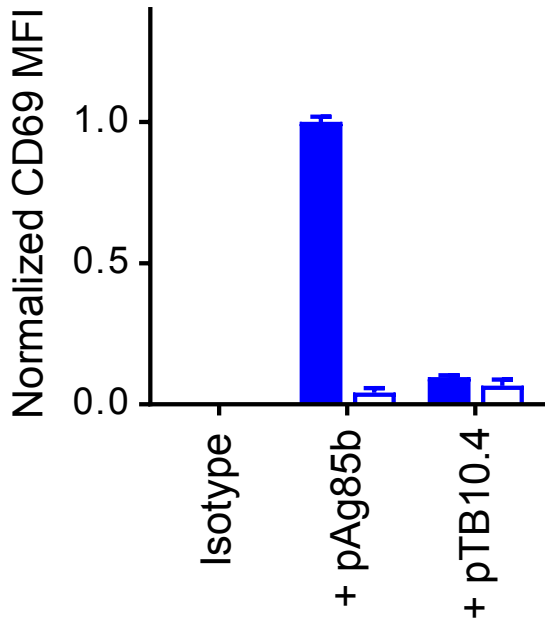

B.

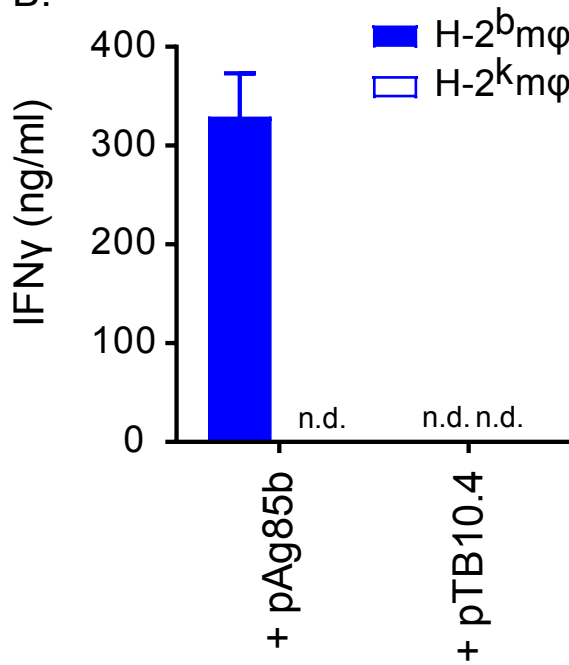

Supplement: S1 Fig — MHC-matched (H-2b) and MHC-mismatched (H-2k) macrophages were pulsed with 10 μM of Ag85b240-254 or TB10.44−11 synthetic peptides. After 1 hour, unbound peptides were washed out, and P25 T cells were added for 72 hours. Induction of CD69 was measured by flow cytometry and IFNγ was measured by ELISA. The normalized CD69 MFI (a) and IFNγ (b) produced by P25 T cells were measured. Data representative of 3 experiments. (PDF) [file ppat.1007060.s001.pdf]

A.

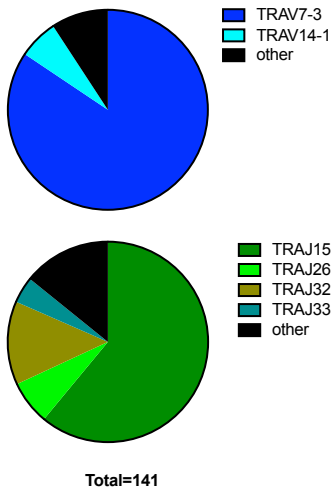

B.

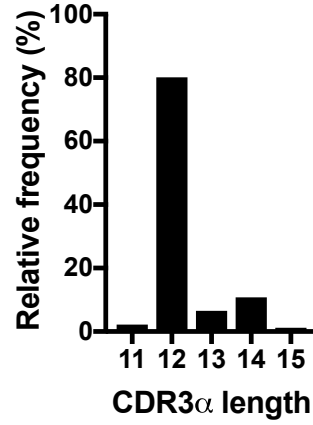

C.

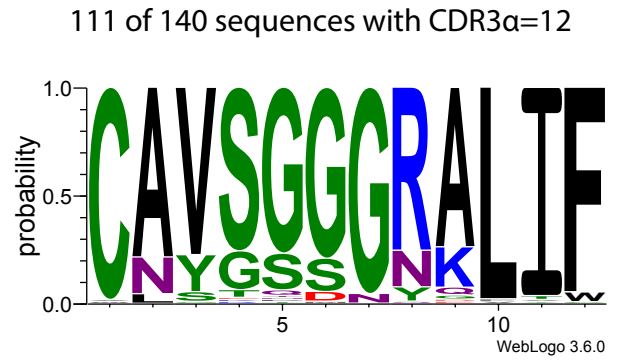

D.

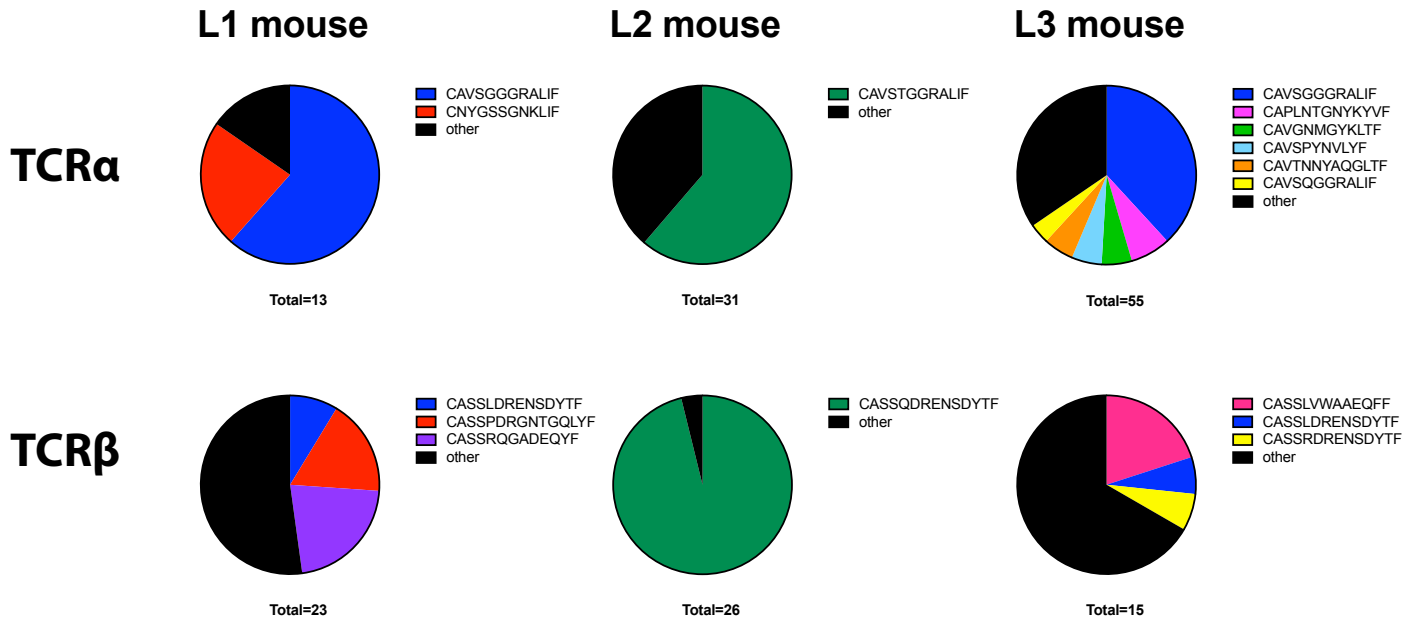

E.

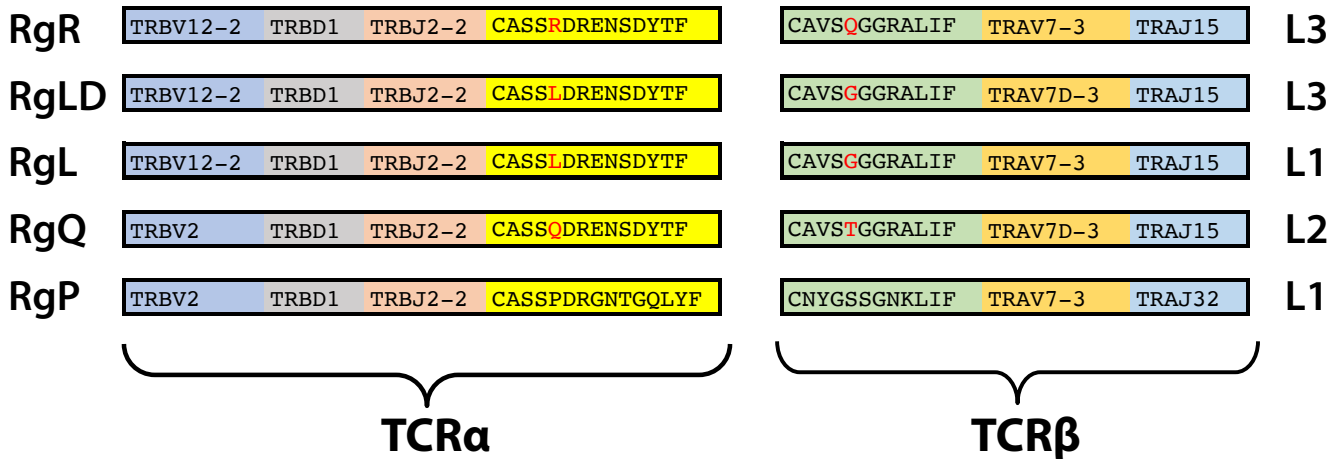

Supplement: S2 Fig — Mononuclear lung cells were obtained from three individual C57BL/6 mice, 9 weeks after low-dose aerosol infection with Mtb Erdman. Single TB10.44−11-tetramer+ CD8+ T cells from each mouse were sorted into 96-well plates and the CDR3α and CDR3β sequences determined as described [20]. The analysis of a representative mouse is shown, for which 141 CDR3α sequences was determined. A clonal expansion of TB10.44−11-tetramer+CD8+ T cells, as previously described [20], was suggested by the skewed distribution of TRAV and TRAJ families (a), which shows an extreme bias in the use of TRAV7 and TRAJ15 gene segments, as well as a dominant CDR3α amino acid (aa) length of 12 (b). (c) Analysis of all CDR3α aa sequence with a length of 12 (n = 112) identify a consensus motif of CAVSGGGRALIF for TB10.44−11-specific CD8+ T cells. Amplification of CDR3α and CDR3β sequences from the same well allowed pairing of TCRα and TCRβ for individual TB10.44−11-specific CD8+ T cells. Three individual mice were analyzed in this manner (d). We identified an expanded CDR3β sequence containing the “xDRENSD” motif, the same motif that had been previously defined by NexGen sequencing [20]. Thus, mouse L1 had an expansion of CD8+ T cells with the CASSLDRENDYTF CDR3β sequence, mouse L2 was dominated by CD8+ T cells using the CDR3β sequence CASSQDRENDYTF, and mouse L3 expressed two major expansions, one encoding CASSLDRENDYTF and the other, CASSDDRENDYTF (d). Based on our ability to pair the CDR3α and CDR3β sequences, we detected an interesting reciprocal conservation. Namely, the ‘xDRENSD’ CDR3β motif was matched to a ‘SxGGRA’ CDR3α motif (e). Finally, we identified an expansion of a T cell clone in mouse L1, which expressed a novel sequence that we had not previously observed (i.e., CASSPDRGNTGQLYF) (d, e). Thus, with a high degree of confidence, we paired the CDR3α and CDR3β sequences belonging to 5 distinct TB10.44−11-specific CD8+ T cell clones that had been expanded in lungs of Mtb-infected C57BL/6 mi [file ppat.1007060.s002.pdf]

A.

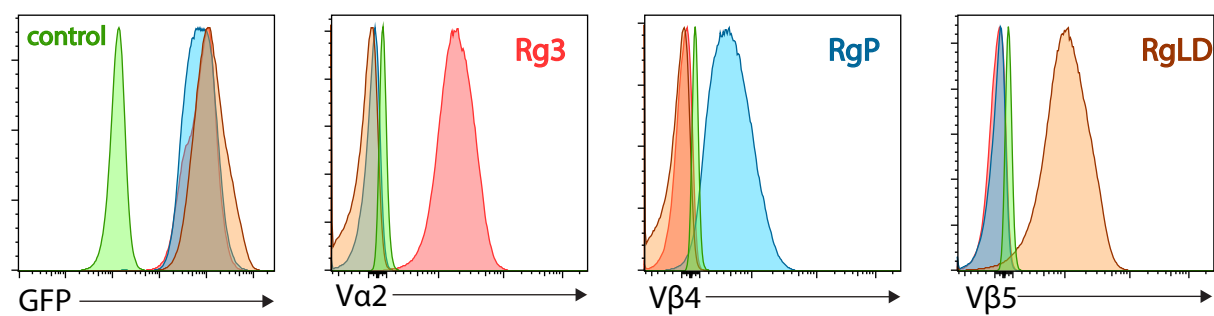

B.

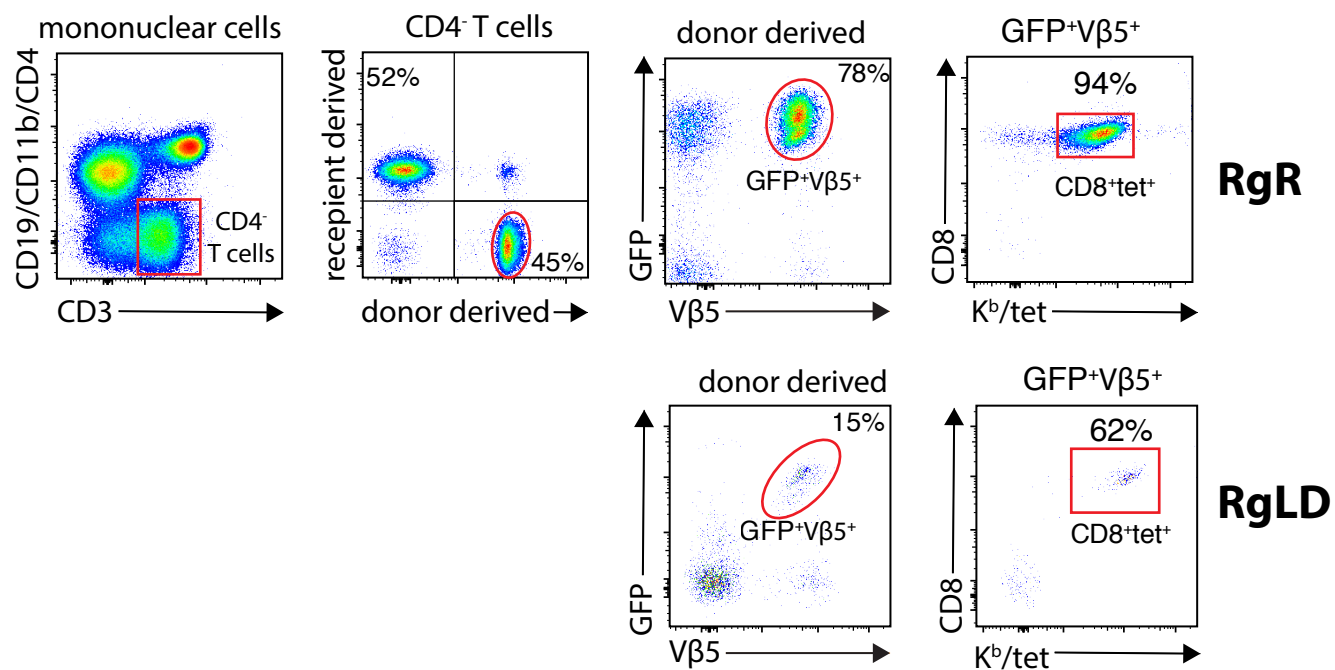

C.

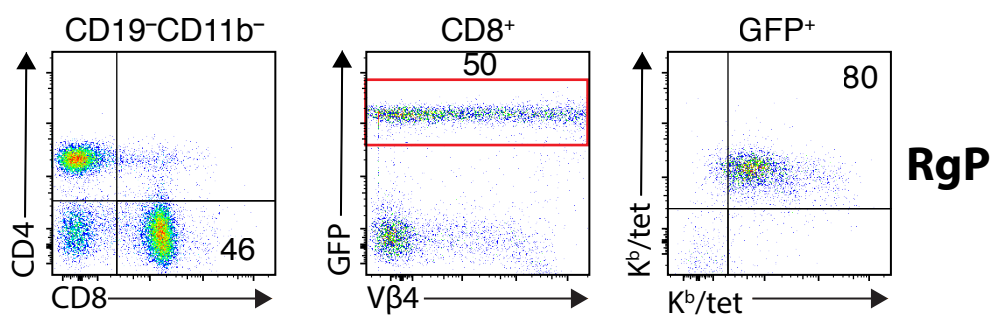

Supplement: S3 Fig — Retrogenic mice were produced as previously described [20]. Six weeks after retroviral transduction of bone marrow and reconstitution of congenically marked recipient mice, the expression of the recombinant TCR was determined in peripheral blood. (a) The BW58α-β- cell line was transduced with different retroviral constructs. GFP+ cells were sorted three times, and mAbs specific for Vα or Vβ were used to confirm successful TCR expression and pairing of TB10RgP and TB10RgLD. The TB10Rg3 construct was included as internal control. (b) Representative flow cytometry plots showed gating strategy of donor-derived GFP+ specific Vβ+ TB10.44−11-tetramer+ CD8+ TB10RgR and TB10RgLD mice. (c) Representative flow cytometry plots of splenic T cells from TB10RgP retrogenic mice demonstrating CD8+GFP+ T cells staining with the TB10.44−11-tetramer. (PDF) [file ppat.1007060.s003.pdf]

S4 Fig

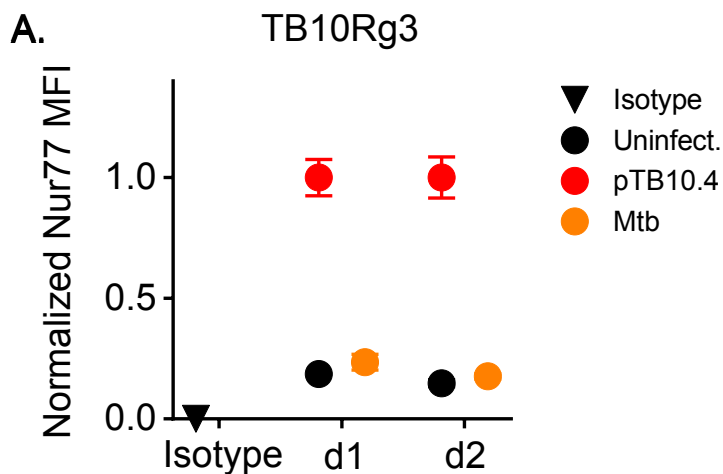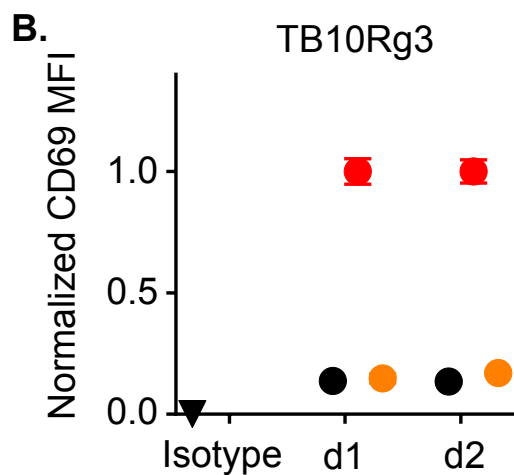

Supplement: S4 Fig — To determine whether a higher MOI would lead to more TB10 antigen production and presentation to TB10Rg3 CD8 T cells, TGPMs were infected with H37Rv at high MOI (average effective MOI of 1.65 to 5.98). TB10Rg3 T cells were added on d1 and d2 post infection for 2 hours, and their expression of Nur77 (a) and CD69 (b) were quantified. Data representative of at least 2 experiments for each time point. (PDF) [file ppat.1007060.s004.pdf]

S5 Fig

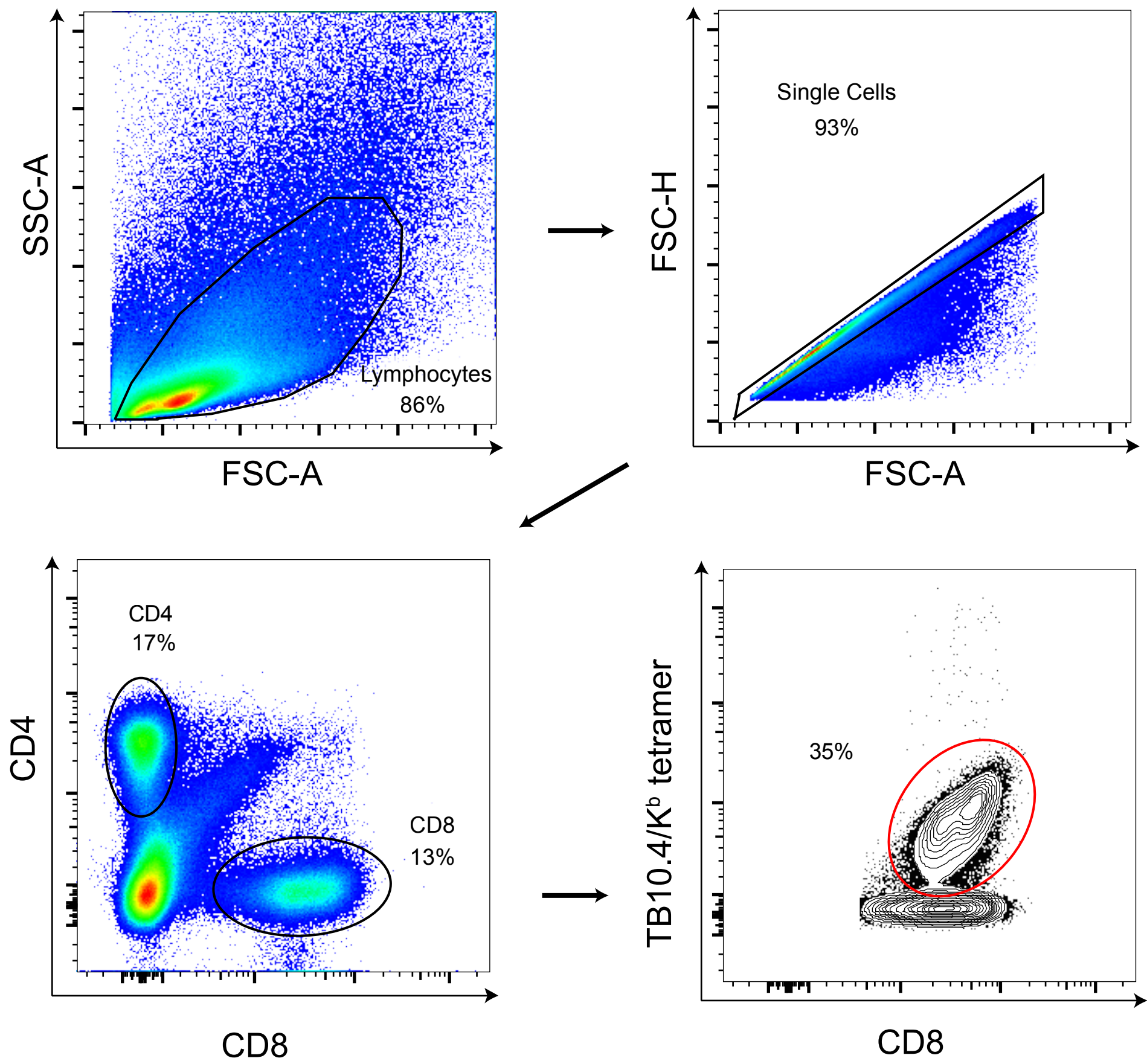

Supplement: S5 Fig — Representative flow plot showing the percent of TB10.44−11-tetramer positive CD8+ T cells among lung cells isolated from mice infected with Mtb Erdman via the aerosol route 6 weeks post-infection. Total lung mononuclear cells were stained with antibodies and tetramers and analyzed by flow cytometry. Lymphocytes were gated based on forward and side scatter and doublets were excluded. CD8 cells were distinguished from CD4 cells. TB10.4-tetramer+ CD8s were identified among the CD8 cell population. (PDF) [file ppat.1007060.s005.pdf]

S6 Fig

Polyclonal CD8<sup>+</sup> T cells

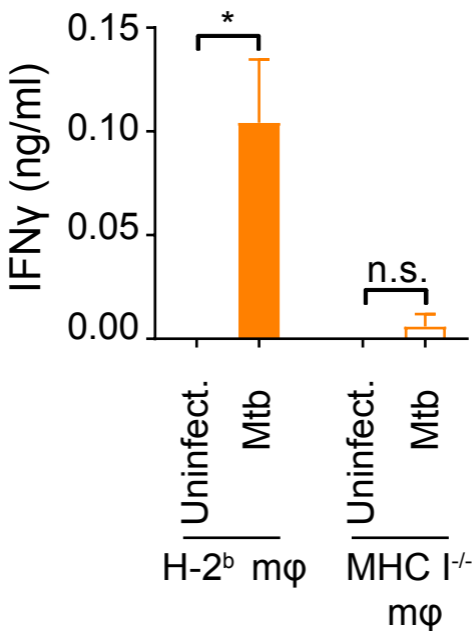

Supplement: S6 Fig — Polyclonal CD8+ T cells were purified from the lungs of C57BL/6J mice, and immediately cultured with either WT (H-2b mφ) or KbDb-/- (MHC I-/- mφ). After 72 hours, IFNγ in the cultures was measured by ELISA. Data is representative of 2 experiments. Statistical testing by a two-tailed, unpaired Student’s T test. *, p<0.05; **, p<0.01; and ***, p<0.005. (PDF) [file ppat.1007060.s006.pdf]
